# Evaluation of blood-brain barrier transport and CNS drug metabolism in diseased and control brain after intravenous L-DOPA in a unilateral rat model of Parkinson's disease

**DOI:** 10.1186/2045-8118-9-4

**Published:** 2012-02-08

**Authors:** Paulien GM Ravenstijn, Henk-Jan Drenth, Michael J O'Neill, Meindert Danhof, Elizabeth CM de Lange

**Affiliations:** 1 Division of Pharmacology, LACDR Leiden University, Leiden, The Netherlands; 2 LAP&P Consultants BV, Leiden, The Netherlands; 3 Neurodegenerative Diseases Drug Hunting Team, Eli Lilly & Co Ltd., Windlesham, UK; 4 LACDR/Pharmacology, Gorlaeus Laboratories, Leiden University, Einsteinweg 55, 2333 CC, Leiden, The Netherlands

**Keywords:** Population pharmacokinetic modelling, Parkinson's disease, rat rotenone model, BBB transport, L-DOPA, microdialysis

## Abstract

**Background:**

Changes in blood-brain barrier (BBB) functionality have been implicated in Parkinson's disease. This study aimed to investigate BBB transport of L-DOPA transport in conjunction with its intra-brain conversion, in both control and diseased cerebral hemispheres in the unilateral rat rotenone model of Parkinson's disease.

**Methods:**

In Lewis rats, at 14 days after unilateral infusion of rotenone into the medial forebrain bundle, L-DOPA was administered intravenously (10, 25 or 50 mg/kg). Serial blood samples and brain striatal microdialysates were analysed for L-DOPA, and the dopamine metabolites DOPAC and HVA. *Ex-vivo *brain tissue was analyzed for changes in tyrosine hydroxylase staining as a biomarker for Parkinson's disease severity. Data were analysed by population pharmacokinetic analysis (NONMEM) to compare BBB transport of L-DOPA in conjunction with the conversion of L-DOPA into DOPAC and HVA, in control and diseased cerebral hemisphere.

**Results:**

Plasma pharmacokinetics of L-DOPA could be described by a 3-compartmental model. In rotenone responders (71%), no difference in L-DOPA BBB transport was found between diseased and control cerebral hemisphere. However, in the diseased compared with the control side, basal microdialysate levels of DOPAC and HVA were substantially lower, whereas following L-DOPA administration their elimination rates were higher.

**Conclusions:**

Parkinson's disease-like pathology, indicated by a huge reduction of tyrosine hydroxylase as well as by substantially reduced levels and higher elimination rates of DOPAC and HVA, does not result in changes in BBB transport of L-DOPA. Taking the results of this study and that of previous ones, it can be concluded that changes in BBB functionality are not a specific characteristic of Parkinson's disease, and cannot account for the decreased benefit of L-DOPA at later stages of Parkinson's disease.

## Background

Tyrosine is usually considered as the starting point in the biosynthesis of dopamine (DA). It is taken up into the brain and subsequently from brain extracellular fluid into dopaminergic neurons where its is converted to 3,4-dihydroxyphenylalanine (L-DOPA), by tyrosine hydroxylase (TH). Aromatic amino acid decarboxylase (AADC) then converts L-dopa to DA and stored in vesicles for neurotransmission [1]. Dopamine is metabolized outside the vesicles where monoamine oxidase (MAO) and aldehyde dehydrogenase transform DA into 3,4-dihydroxyphenylacetic acid (DOPAC) which then diffuses out of the cells. Subsequently, DOPAC is mainly transformed to homovanillic acid (HVA) by catechol-O-methyltransferase (COMT) [2,3].

It is known that in Parkinson's disease dopaminergic neurons in the nigro-striatal pathway are progressively damaged [4], which causes a decrease in dopamine concentration in the striatum. Current therapy for Parkinson's disease focuses mainly on symptomatic treatment to replace the lost dopamine in the striatum. The drug that is routinely used for the symptomatic treatment of Parkinsonism is L-3,4-dihydroxyphenylalanine also known as L-DOPA or levodopa [5]. For patients with early-stage Parkinson's disease, the treatment with L-DOPA is quite successful. However, the benefits of this drug gradually decline in the later stages [4-9]. It may be that this decreased benefit of L-DOPA is solely due to a reduction in the number of viable dopaminergic neurons that can convert L-DOPA into dopamine to reduce the symptoms. However, it may also be that the pharmacokinetics of L-DOPA in the brain for the same dose of L-DOPA may change during disease progression, due to alterations in the functionality of the blood-brain barrier (BBB) [10].

The question whether the BBB is affected in Parkinson's disease is still a matter of debate. Results from different investigations in animal models or patients with Parkinson's disease vary. Carvey *et al*. [11] observed areas of BBB leakage in 6-OH-dopamine-treated rats, in an acute model for Parkinson's disease, using FITC-labelled albumin and horseradish peroxidase.

Furthermore, they found areas with increased expression of P-glycoprotein (P-gp), and showed that the dopamine antagonist domperidone, that normally has highly limited brain distribution due to its high affinity for P-gp, was able to attenuate apomorphine-induced stereotypic behaviour in these animals. In human positron emission tomography (PET) studies, Bartels *et al*. found that P-glycoprotein exhibits decreased function in patients with advanced but not early Parkinson's disease [12,13]. The authors suggested that breakdown of the BBB may occur with increasing severity of the disease. In contrast, in the unilateral rat rotenone model for progressive Parkinson's disease, no changes in BBB transport were found for fluorescein [14]. Also, in the primate brain, Astradson, *et al*. [15] found no disruption of the BBB using *in vivo *neuroimaging techniques with gadolinium-diethylenetriamine pentaacetic acid (Gd-DTPA). With regard to BBB transport for L-DOPA being dependent on the L-type amino acid influx transporter 1 (LAT1) [16], it is of interest that Ohtsuki *et al*. [17] found a ~50% reduction of LAT1 mRNA expression at the BBB in mice, 7 days after treatment with 1-methyl-4-phenyl-1,2,3,6-tetrahydropyridine (MPTP), in conjunction with motor deficits and a loss of dopaminergic neurons. Also, Westin *et al*. [18] found BBB defects in the basal ganglia in MPTP lesioned/chronic L-DOPA-treated animal models of Parkinson's disease. Finally, Alexander *et al*. [19] found an inverse relationship between the ability of MPTP-treated monkeys to transport L-DOPA from blood to brain and their degree of Parkinsonism.

Studies on the potential *mutual *interactions of BBB transport, the fate of L-DOPA metabolism in the brain, and the number of viable dopaminergic neurons under normal and diseased conditions are needed for improved understanding of Parkinson's disease. In this study using a unilateral rat model of Parkinson's disease, we characterized the BBB transport of L-DOPA, L-DOPA's neuropharmacokinetics and its associated conversion, via dopamine, into the main dopamine metabolites DOPAC and HVA, in healthy as well as in late-stage diseased brain. Our hypothesis was that a decrease in BBB transport of L-DOPA would contribute to the decline in L-DOPA efficacy at later stages of Parkinson's disease.

In this study, intracerebral microdialysis was used for the determination of BBB transport of L-DOPA [20]. At 14 days after unilateral rotenone infusion into the medial forebrain bundle (MFB), microdialysate samples were collected in parallel with serial blood samples. Concentrations of L-DOPA, dopamine, DOPAC and HVA concentrations were determined in the striatal microdialysis samples, from both the rotenone-infused and the control side. These concentrations were determined under basal conditions as well as following intravenous administration of 10, 25 or 50 mg/kg of L-DOPA. At the end of the experiment, brains were removed for TH immunostaining to determine responders and non-responders, from the percentage loss of dopaminergic terminals in the striatum. The mutual interactions between the different processes were determined by developing a mathematical model on the basis of the interconnected data obtained.

## Methods

### Animals and surgical procedures

#### Animals

All animal procedures described in this paper were approved by the Ethical Committee on Animal Experimentation of the University of Leiden (DEC numbers 118 and 5069). Experiments were performed on male Lewis rats (Charles River BV, Maastricht, The Netherlands) weighing 311 ± 17 g (mean ± s.d., n = 18) and 288 ± 13 g (mean ± s.d., n = 17) before surgery and before start of the experiment, respectively. The rats were housed in standard plastic cages (six per cage before surgery and individually after surgery) with a 12-hour day/night schedule (lights on 7:30 a.m.) and at a temperature of 21°C. The animals had access to standard laboratory chow (RMH-TM; Hope Farms, Woerden, The Netherlands) and acidified water *ad libitum*.

#### Surgery

The surgery for the microdialysis study was performed under anesthesia with an intramuscular injection of 0.1 mg/kg medetomidine hydrochloride (Domitor 1 mg/ml, Pfizer, Capelle a/d IJssel, The Netherlands) and 1 mg/kg ketamine base (Ketalar 50 mg/ml, Parke-Davis, Hoofddorp, The Netherlands). Three indwelling cannulae (pyrogen-free, nonsterile polyethylene tubing, Portex Limited) were implanted, one in the left femoral artery for blood sampling and two in the left femoral vein for drug administration. The cannulae were tunnelled subcutaneously and fixed at the back of the neck with a rubber ring. The skin in the neck was stitched with normal sutures. The skin in the groin was closed with wound clips. To prevent clotting and obstruction, the cannulae were filled with a 25% (w/v) polyvinylpyrrolidone solution (PVP; Brocacef, Maarssen, The Netherlands) in pyrogen-free physiological saline (B. Braun Melsungen AG, Melsungen, Germany) containing 20 IU/ml heparin (Hospital Pharmacy, Leiden University Medical Center, Leiden, The Netherlands).

After the implantation of the intravascular cannulae, the rats were placed in a stereotaxic frame and the skull was exposed for brain surgery. The skull was cleaned and a hole was drilled to allow a needle (30 G, Microlance, Becton Dickinson) to be lowered into the right MFB using the co-ordiantes AP: -2.8; L: +2.0; V: -9.0 relative to bregma [21] for unilateral infusion of 5.0 μg of rotenone (Rotenone Pestanal^® ^Sigma Alldrich BV, Zwijndrecht, the Netherlands) at a rate of 0.1 μl/min for 30 min. Rotenone was dissolved in a 1:1-mixture of dimethylsulfoxide (DMSO, Sigma Alldrich BV) with polyethylene glycol (PEG 200, Sigma Alldrich BV). After the infusion, the needle was kept in place for another 5 min to prevent leakage along the track of the needle. Subsequently, two small holes were drilled into the skull to allow implantation of the microdialysis guide cannulae (CMA/12, Aurora Borealis Control BV, Schoonebeek, The Netherlands) in the left and in the right striatum, AP: +0.4; L: +/-3.2; V: -3.5 relative to bregma [21]. One support screw was placed as an extra anchor for the guide, which was glued to the skull with dental acrylic cement (How media simplex rapid + methylacrylate, Drijfhout, Amsterdam, The Netherlands). After surgery, the rats were allowed to recover for 13 days, after which they were randomly assigned to one of three dosing groups of 10 mg/kg (n = 4); 25 mg/kg (n = 4); and 50 mg/kg (n = 5) of L-DOPA. The intracerebral unilateral rat rotenone model used in these experiments, has been described previously by our group [14].

### Microdialysis experimental setup

At 13 days after the unilateral infusion of rotenone into the right MFB, and 18-24 hours prior to the experiment, the microdialysis probes (CMA12, membrane length of 4.0 mm; Aurora Borealis Control BV) were inserted into the guide cannulae. All animals were fasted overnight prior to the experiment in order to rule out any competition in BBB transport of L-DOPA with food-related amino acids [22]. The microdialysis experiment, 14 days post rotenone treatment, was started between 7:00 and 8:00 a.m. The inlets of the microdialysis probes were connected by FEP tubing (fluorinated ethylene propylene tubing; Aurora Borealis Control BV) to syringe pumps (Beehive, Bas Technicol, Congleton, United Kingdom). The probes were perfused with artificial ECF (composition in mM: NaCl 145; KCl 2.7; CaCl_2 _1.2; MgCl_2 _1.0; ascorbic acid 0.2 in a 2 mM phosphate buffer pH 7.4) [23] at a flow rate of 2 μl/min. The outlets also consisted of FEP tubing and were connected to a microsample collector (Univentor 820; Antec, Leiden, The Netherlands), in which the samples were cooled (4°C). The vials contained an antioxidant fluid (0.1 M acetic acid, 3.3 mM L-cysteine, 0.27 M EDTA, 0.0125 mM ascorbic acid dissolved in Millipore water) in a ratio of 1:4 with the expected volume of the microdialysis sample, to prevent the breakdown of the catecholamines.

After a stabilisation period of 60 min, the *in vivo *recovery of L-DOPA was determined by the retrodialysis method. For this purpose, the probes were first perfused with a L-DOPA solution (10, 100 or 200 ng/ml in the perfusion fluid for the 10, 25 and 50 mg/kg dose groups, respectively) for 60 min to collect 6 fractions. The relative loss of L-DOPA was determined and used for estimating brain extracellular fluid (brain_ECF_) concentrations. After this period, the syringes were switched to blank perfusion fluid for the washout phase of 90 min.

After the washout period, the intravenous administration of L-DOPA was started. One venous cannula was connected to a syringe containing L-DOPA (Sigma Alldrich BV, Zwijndrecht, the Netherlands) in 0.2 M HCl in saline (0.9% NaCl) and ascorbic acid (5% of the L-DOPA amount) and the second venous cannula was connected to a syringe containing 7% NaHCO_3 _to neutralize the acidic L-DOPA solution. Both infusions were started at the same time for 20 min at a rate of 20 μl/min. In the first 120 min of the experiment, microdialysis fractions were collected at 10-min intervals. From 120-180 min, microdialysis fractions were collected at 20-min intervals. From 180 min until the end of the experiment (at 360 min), microdialysis fractions were collected at 30-min intervals. Blood samples (50 μl in heparinised Eppendorf vials) were taken at pre-dose (5 min before), and at 5, 10, 15, 20, 22, 24, 26, 28, 30, 45, 50, 60, 75, 90, 120, 180, 240, 360 min after start of the L-DOPA infusion. The blood samples were centrifuged for 10 min at 5000 rpm and the plasma was pipetted into Eppendorf vials. All samples were stored at - 80°C before analysis. After the experiment, the animals were given an overdose of sodium pentobarbital (Nembutal, Ceva Santa Animale, Maassluis, The Netherlands) and the thorax was opened and the vascular bed was perfused via the left ventricle of the heart with 30 ml of saline followed by 30 ml of 10% phosphate buffered formalin (pH 7.0). Brains were removed for histopathology.

### Immunohistopathology

TH immunohistochemistry was performed to quantify the degree of dopaminergic depletion as described previously [14]. After staining, striatal sections were analysed by measuring the optical density of manually-defined areas on each black and white image produced as a mean grey value (MGV). Optical density was measured for the slide (background), cortex (control tissue staining), corpus callosum (control non-cellular staining), dorsal striatum (caudate putamen, CPu) and ventral striatum (nucleus accumbens, NAcc). The values were adjusted for non-specific staining by subtracting the MGV of the corpus callosum or cortex. Mean, standard deviation and standard error of the mean (sem) of MGV was calculated. All striatal MGV values are corrected for cortical MGV. The percentage of intact TH staining in the rotenone-treated hemisphere was calculated as the percentage of striatal MGV compared to the striatal MGV of the untreated hemisphere. In the clinical setting, symptoms of Parkinson's disease arise when about 80% of striatal dopamine and about 60% of dopamine neurons are lost [4]. The rats which exhibited a TH staining level lower than 40% (60-100% of the dopamine terminals were lost) were considered as 'responders' to the rotenone treatment.

### Analysis of L-DOPA, DOPAC and HVA

All plasma samples were analysed for L-DOPA and all microdialysate samples were analysed for L-DOPA, dopamine, DOPAC and HVA using a high performance liquid chromatography (HPLC) system with electrochemical detection (ECD).

#### HPLC and Electrochemical Detection system

The HPLC system consisted of a LC-10AD HPLC pump (Shimadzu, 's Hertogenbosch, The Netherlands), a Waters 717 Plus autosampler (Waters, Etten-Leur, The Netherlands), a pulse damper (Antec Leyden, Zoeterwoude, The Netherlands) and a digital electrochemical amperometric detector (DECADE, software version 3.02, Antec Leyden). The electrochemical detector consisted of a VT-03 electrochemical flow cell combined with a 25 μm spacer and an *in situ *Ag/AgCl (ISAAC) reference electrode operating in the DC mode. For the analysis, a standard Ag/AgCl reference electrode, filled with a saturated KCl solution was used. Data acquisition and processing was performed using the Empower^® ^data-acquisition software (Waters).

#### Analysis of L-DOPA in plasma

Chromatography of plasma samples of L-DOPA was performed on a Beckman Coulter™ Ultrasphere^® ^5 μm C-18 column (4.6 mm I.D. × 150 mm, Alltech, Breda, The Netherlands) equipped with a refill guard column (2 mm I.D. × 20 mm, Upchurch Scientific, Oak Harbor, WA, USA) packed with pellicular C18 material (particle size 20-40 μm, Alltech) at a constant temperature of 30°C. The mobile phase was a mixture of 0.05 M sodium phosphate buffer (pH 2.8) and methanol (90:10, v/v), supplemented with 0.3 mM EDTA (sodium salt) and 10 mM octane-sulfonic acid. Before the addition of methanol, the mobile phase was filtered through a 0.2 μm nylon filter (Alltech), then the methanol was added and it was mixed and degassed with helium. The flow rate was set at 1 mL/min. The optimal working potential for L-DOPA was +0.75 V, as determined by a voltammogram and sensitivity plot. Concentrations were measured at a sensitivity range of 5 nA for L-DOPA and 20 nA for 3,4-dihydroxybenzylamine hydrobromide (DHBA; internal standard). Stock solutions of L-DOPA were prepared at a concentration of 1 mg/mL in Millipore water. The stock solutions were diluted with Millipore water to obtain calibration solutions in the range of 2 to100 ng/mL. The internal standard (DHBA) solution was prepared by dilution of the stock solution to a final concentration of 500 ng/mL. The stock solutions were stored at -80°C for up to one month. The assay solutions were prepared freshly before each analysis. For determination of the L-DOPA in plasma, 25 μL of internal standard solution (DHBA 500 ng/mL) was added to 45 μL plasma samples and 50 μL of Millipore water in glass centrifuge tubes. Next, 25 μL of 20% TCA was added and the mixture was vortexed for 5 min. After centrifugation for 10 min at 4000 rpm (2000 g), 100 μL of the supernatant was added to 50 μL of phosphoric buffer (1 M, pH 5.5) of which 25 μL was injected into the HPLC system.

#### Analysis of L-DOPA, dopamine, DOPAC and HVA in microdialysate

For analysis of L-DOPA, dopamine, DOPAC and HVA brain microdialysate concentrations, 5 μL of internal standard (isoproterenol, 100 ng/mL) solution was added per 10 μL of microdialysis sample or calibration curve sample. The samples were then injected (20 μL) into the HPLC system without further sample pre-treatment. Chromatography of brain microdialysate samples was performed on a Beckman Coulter™ Ultrasphere^® ^5 μm C-18 column (2 mm I.D. × 250 mm, Alltech) at a constant temperature of 30°C. The mobile phase was a mixture of 0.05 M sodium phosphate buffer (pH 2.8) and methanol (88:12, v/v), supplemented with 0.3 mM EDTA (sodium salt) and 1.5 mM octane-sulfonic acid. Mobile phase solvents were filtered through a 0.2 μm nylon filter. The methanol was added and the mobile phase was mixed and degassed with helium. The flow rate was set at 0.2 mL/min. The optimal working potential for a mixture of L-DOPA, dopamine, DOPAC and HVA was +0.66 V, as determined by a voltammogram and sensitivity plot. Concentrations were measured at a sensitivity range of: 0.1 nA for dopamine, 0.5 nA for L-DOPA, HVA and isoproterenol; and 10 nA for DOPAC. For analysis of brain microdialysate samples, stock solutions of L-DOPA, DOPAC and HVA were prepared at a concentration of 0.5 μg/mL for dopamine, 1 μg/mL for L-DOPA and 5 μg/mL for DOPAC and HVA in microdialysis perfusion fluid with aqueous antioxidant solution consisting of 0.1 M acetic acid, 3.3 mM L-cysteine, 0.27 M EDTA (sodium salt) and 0.0125 mM ascorbic acid (4:1 v/v). Internal standard solution was freshly prepared before each analysis by dilution of a 1 mg/mL isoproterenol stock solution, to a concentration of 100 ng/mL of the compound in perfusion fluid that contains antioxidant (4:1 v/v). All the stock solutions were stored at -80°C for up to one month. Before each analysis a first calibration solution containing all compounds was freshly prepared by mixing one volume of each stock solution (L-DOPA, dopamine, DOPAC and HVA) and adding 5 volumes of perfusion fluid with antioxidant (6:1 v/v). This first calibration solution now contained all compounds at a concentration 10 times lower than their stock solution and from this solution the other calibration solutions were prepared.

### Population pharmacokinetic data analysis- model development

The pharmacokinetics of L-DOPA, DOPAC and HVA were analysed utilizing a population pharmacokinetic modelling approach. Dopamine concentrations were all below the limit of detection. Compartmental modelling was performed using the ADVAN6 subroutine in NONMEM VI release 2 (GloboMax LLC, Hanover, MD, USA). All fitting procedures were performed on an IBM-compatible computer (Pentium IV, 1500 MHz) running under Windows XP with the Compaq Visual Fortran compiler version 6.6. The inter-individual variability of model parameters was described by an exponential equation, according to:

$${\text{P}}_{\text{1i}}=\theta_{\text{1}}\cdot \text{exp}\left(\eta_{\text{i}}\right),$$

where θ_1 _is the population (typical) estimate for parameter P_1_, P_1i _is the individual estimate and η_i _determines the random deviation of P_1i _from P_1_. The values of η_i _are assumed to be randomly, normally distributed with mean zero and variance ω_11_^2^. The residual error in the L-DOPA concentration was described by a proportional error model:

$${\text{C}}_{\text{obs},\text{ij}}={\text{C}}_{\text{pred},\text{ij}}\;\cdot \text{ (1}+\varepsilon_{\text{ij}}),$$

and the residual error in the DOPAC or HVA concentration was described by an additive error model:

$${\text{C}}_{\text{obs},\text{ij}}={\text{C}}_{\text{pred},\text{ij}}+\varepsilon_{\text{ij}}$$

where C_obs, ij _represents the j^th ^measured L-DOPA, DOPAC or HVA concentration for the i^th ^individual predicted by the model. C_pred, ij _represents the prediction of concentration and ε_ij _is the deviation of the model-predicted value from the observed concentration. The values of ε are assumed to be randomly, normally distributed with mean zero and variance σ^2^.

The first order conditional estimation method with interaction (FOCE interaction) was used in NONMEM to fit the models to the data and to estimate θ's, ω^2^'s and σ^2^'s. Structural model selection for all models was based on the likelihood ratio test, diagnostic plots (observed concentrations vs. individual and population predicted concentrations, conditional weighted residuals vs. time and predicted concentrations), parameter correlations and precision in parameter estimates. Inclusion of one parameter into the model was assumed to be significant if this led to a decrease of 10.8 points or more of the minimum value of the objective function (MVOF) after fitting the model to the data. This corresponds to a theoretical significance level of *p *= 0.001 under the assumption that the difference in MVOF between two nested models is χ^2 ^distributed.

In total, the L-DOPA plasma profiles of 13 rats (10 mg/kg, n = 4; 25 mg/kg, n = 4; 50 mg/kg, n = 5), the L-DOPA brain_ECF _profiles from the control cerebral hemisphere of 12 rats (10 mg/kg, n = 4; 25 mg/kg, n = 3; 50 mg/kg, n = 5) and the L-DOPA brain_ECF _profiles from the rotenone-treated responder cerebral hemisphere of 7 responder rats (10 mg/kg, n = 1; 25 mg/kg, n = 2; 50 mg/kg, n = 4) were included in the population pharmacokinetic analysis.

**Figure 1:**
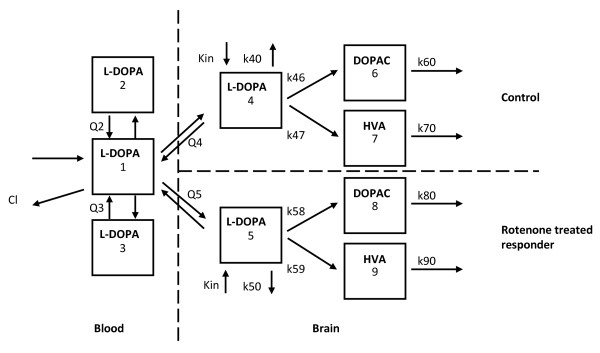
**The population pharmacokinetic model for L-DOPA, DOPAC and HVA comprising of three compartments (1-3) describing the pharmacokinetics of L-DOPA in plasma, two compartments (4 and 5) describing the pharmacokinetics of L-DOPA in brain_ECF_, one for the control cerebral hemisphere and one for the rotenone-treated responder cerebral hemisphere, two compartments (6 and 8) describing the kinetics of DOPAC in brain_ECF_, one for the control cerebral hemisphere and one for the rotenone-treated responder cerebral hemisphere and two compartments (7 and 9) describing the kinetics of HVA in brain_ECF_, one for the control cerebral hemisphere and one for the rotenone-treated responder cerebral hemisphere**. (V = volume of distribution, Q = inter-compartmental clearance, k = elimination rate constant, Kin = endogenous formation rate constant of L-DOPA.).

On the basis of selection criteria, the plasma and brain_ECF _L-DOPA data from the individual rats were simultaneously analysed (Figure 1; compartments 1-5). First, clearances from compartment 1 to 4 (Cl14), compartment 4 to 1 (Cl41), compartment 1 to 5 (Cl15) and compartment 5 to 1 (Cl51) were assigned in the structural model to seek for concentration-dependent BBB transport of L-DOPA via the LAT-1 transporter. However, although a large range of plasma concentration data was available (3 dosages of L-DOPA), no asymmetry in BBB transport could be identified. The model was therefore simplified to use the inter-compartmental clearances Q4 and Q5.

Individual model parameters (*post hoc *estimates) for each rat from this analysis and typical values from this model (Table 1) were then used as input for the subsequent analysis of DOPAC (Figure 1; compartments 6 and 8) and HVA (Figure 1; compartments 7 and 9). In total, the DOPAC and HVA microdialysate concentrations obtained from the control cerebral hemisphere of 12 rats (10 mg/kg: n = 4; 25 mg/kg: n = 3; 50 mg/kg: n = 5) and the DOPAC and HVA microdialysate concentrations from the rotenone-treated responder cerebral hemisphere of 8 rats (10 mg/kg: n = 2; 25 mg/kg: n = 2; 50 mg/kg: n = 4) were included in the population pharmacokinetic analysis.

**Table 1:** Population pharmacokinetic parameter estimates with the corresponding inter-individual coefficient of variation (CV%) and lower -and upper limit confidence intervals (LLCI and ULCI).

Parameter	Estimate	CV%	LLCI-ULCI
L-DOPA			

Cl (mL/min)	30	21	17 - 43
ω^2 ^Cl	0.26	59	-0.041 - 0.56
V1 (mL)	98	39	24 - 172
V2 (mL)	157	15	112 - 202
V3 (mL)	599	15	425 - 773
V4 (mL)	13300	25	6810 - 19800
ω^2 ^V4	0.075	40	0.020 - 0.13
Q2 (mL/min)	22	24	11 - 32
Q3 (mL/min)	11	16	7.5 - 15
Q4 (mL/min)	22	14	16 - 29
K_in _(min^-1^)	5.8	36	1.7 - 9.8
ω^2 ^K_in_	0.94	41	0.19 - 1.7
Proportional error (plasma)	0.087	15	0.061 - 0.113
Proportional error (ECF)	0.17	21	0.10 - 0.24

Control cerebral hemisphere			
DOPAC			

Formation k46 (min^-1^)	0.000044	28	0.000020 - 0.000068
ω^2 ^k46	0.50	60	-0.084 - 1.1
k40 (min^-1^)	0.53	23	0.29 - 0.77
ω^2 ^k40	0.42	46	0.045 - 0.80
Elimination k60 (min^-1^)	0.0053	17	0.0035 - 0.0071
ω^2 ^k60	0.19	58	-0.025 - 0.41
Residual error (additive)	0.0020	16	0.0014 - 0.0026

HVA			

Formation k47 (min^-1^)	0.000023	15	0.000016 - 0.000030
ω^2 ^k47	0.019	70	-0.0070 - 0.000030
k40 (min^-1^)	0.19	16	0.13 - 0.25
k70 (min^-1^)	0.0044	12	0.0033 - 0.0054
ω^2 ^k70	0.14	53	-0.006 - 0.28
Residual error (additive)	0.0028	24	0.0015 - 0.0041

Rotenone-treated responder cerebral hemisphere			
DOPAC			

Formation k58(min^-1^)	0.000054	51	-0.00000039 - 0.000011
ω^2 ^k58	1.0	40	0.23 - 1.9
k50 (min^-1^)	0.36	55	-0.025 - 0.75
ω^2 ^k50	0.53	36	0.16 - 0.90
Elimination k80 (min^-1^)	0.038	18	0.024 - 0.052
Residual error (additive)	0.0014	51	0.0000020 - 0.0027

HVA			

Formation k59(min^-1^)	0.000016	42	0.0000028 - 0.000030
ω^2 ^k59	0.49	65	-0.14 - 1.12
k50(min^-1^)	0.14	55	-0.010 - 0.29
Elimination k90 (min^-1^)	0.011	39	0.0027 - 0.020
Residual error (additive)	0.0034	41	0.00060 - 0.0061

V = volume of distribution, Q = inter-compartmental clearance, k = elimination rate constant. Note: V5 = V4, Q5 = Q4 and k50 = k40.

## Results

The percentage of intact TH staining in the rotenone-treated cerebral hemisphere compared to the untreated hemisphere at the level of the striatum was below 40% in 12 out of 17 rats (responders), and higher than 90% in the remaining 5 rats (non-responders). No quantifiable concentrations of dopamine could be detected throughout the experiment (limit of quantification was 0.01 ng/mL in a 20 μl microdialysate sample). A total of 17 rats were used in the microdialysis experiments: n = 6 in the 10- and 25 mg/kg dose group and n = 5 in the 50 mg/kg dose group, where for one rat the microdialysis probe malfunctioned. All microdialysate concentrations of L-DOPA were corrected for the average *in vivo *recovery as determined during the retrodialysis period (30 ± 6%) in order to estimate brain_ECF _concentrations. The *in vivo *recovery was equal for the three concentrations of L-DOPA used, and not affected by disease condition.

### Data analysis

#### L-DOPA pharmacokinetic modeling

**Figure 2:**
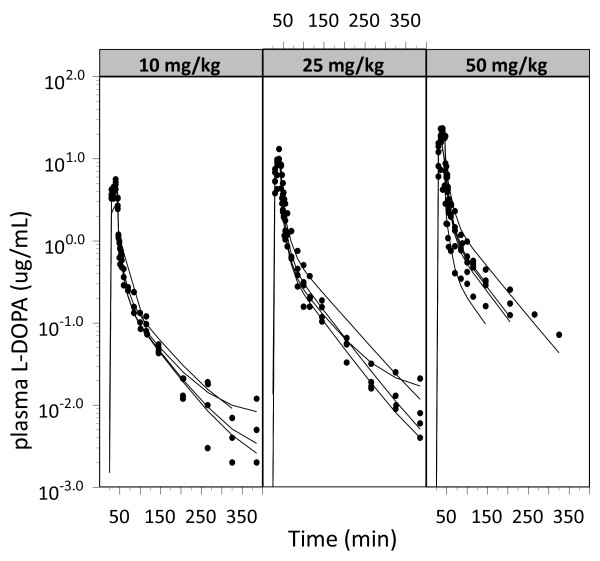
**L-DOPA concentration-time profiles in plasma, obtained after a 20-min intravenous infusion in Lewis rats**. Depicted are the observed concentrations (dots) and individual predictions (solid lines), separated by L-DOPA dose (in total 13 rats: 10 mg/kg, n = 4; 25 mg/kg, n = 4; 50 mg/kg, n = 5).

**Figure 3:**
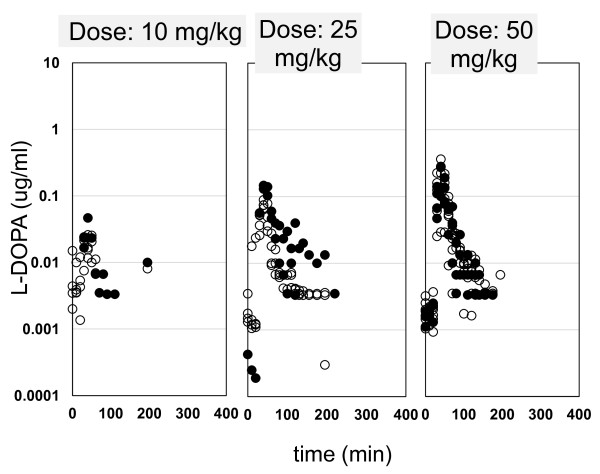
**L-DOPA concentration-time profiles in brain_ECF _in the control cerebral hemisphere (○; in total 12 rats: 10 mg/kg, n = 4; 25 mg/kg, n = 3; 50 mg/kg, n = 5) and in the rotenone-treated responder cerebral hemisphere (•; in total 7 rats: 10 mg/kg, n = 1; 25 mg/kg, n = 2; 50 mg/kg, n = 4), obtained after a 20-min intravenous infusion in Lewis rats**.

The plasma concentration-time profiles for each rat following intravenous infusion of 10, 25, and 50 mg/kg of L-DOPA are shown in Figure 2. The resulting brain_ECF _concentration-time profiles of L-DOPA are shown in Figure 3, for both the control and the responder cerebral hemispheres. The L-DOPA concentrations in plasma and in brain_ECF _were analysed simultaneously. All structural parameters of the population pharmacokinetic model for L-DOPA could be adequately estimated (Table 1). No dose-dependency was observed in the plasma pharmacokinetics for L-DOPA. No significant difference could be detected between the inter-compartmental clearances Q4 and Q5, the volumes of distribution V4 and V5 or the elimination rate constants k40 and k50 when these models were fitted to the L-DOPA brain concentration data. Table 2 shows a summary of the MVOF and parameter estimates after the different assumptions (Q4 = Q5, V4 = V5, k40 = k50 or a combination of any of these). Also, the separate estimate of endogenous brain production rate of L-DOPA (K_in_) for compartment 4 and 5 resulted in similar estimated values (5.8 min^-1^). Striatal brain_ECF _baseline L-DOPA levels in the rotenone-treated responder cerebral hemisphere were not statistically different (*p *= 0.07; Welch's t-test) from those in the control hemisphere and averaged 0.010 ± 0.004 pmol/mL and 0.024 ± 0.011 pmol/mL (mean ± SEM), respectively. Therefore, it can be concluded that no significant difference can be identified between the pharmacokinetics of L-DOPA in the control *versus *the rotenone-treated responder cerebral hemisphere.

**Table 2:** Summary of goodness-of-fit based on the minimum value of objective function (MVOF), of eight assumptions within the modeling of L-DOPA in plasma and brain_ECF_.

Assumption	Results	*MVOF *	V4	V5	Q4	Q5	K40	K50
NONE	general	*-2814*	11100	16100	20,9	25,3	0,211	0,135
	SE		3840	6970	3,74	9,24	0,033	0,0132
	CV(%)		34,6	43,3	17,9	36,5	15,6	9,78

V5 = V4	general	*-2808*	12700		22,9	20,1	0,193	0,161
	SE		5700		4,33	7,13	0,0329	0,0185
	CV(%)		44,9		18,9	35,5	17	11,5

k50 = k40	general	*-2803*	13200	12000	20,1	26	0,175	
	SE		3870	4990	3,36	8,77	0,0226	
	CV(%)		29,3	41,6	16,7	33,7	12,9	

Q5 = Q4	general	*-2813*	11400	14400	22		0,211	0,135
	SE		3080	3700	3,18		0,027	0,0127
	CV(%)		27	25,7	14,5		12,8	9,41

V5 = V4 & k50 = k40	general	*-2804*	13000		21,4	24,3	0,179	
	SE		3410		3,2	5,56	0,0199	
	CV(%)		26,2		15	22,9	11,1	

V5 = V4 & Q5 = Q4	general	*m.t*.	13000		22		0,19	0,17
	SE		-	-	-	-	-	-
	CV(%)		-	-	-	-	-	-

k50 = k40 & Q5 = Q4	general	*-2801*	13900	10600	21,7		0,173	
	SE		8290	6610	7,79		0,0276	
	CV(%)		59,6	62,4	35,9		16	

V5 = V4 & k50 = k40 & Q5 = Q4	general	*-2801*	13300		22,4		0,175	
	SE		3310		3,17		0,0201	
	CV(%)		24,9		14,2		11,5	

The assumption 'NONE' is where all parameters were estimated.

V = volume of distribution, Q = inter-compartmental clearance, k = elimination rate constant

#### DOPAC and HVA kinetic modeling

**Figure 4:**
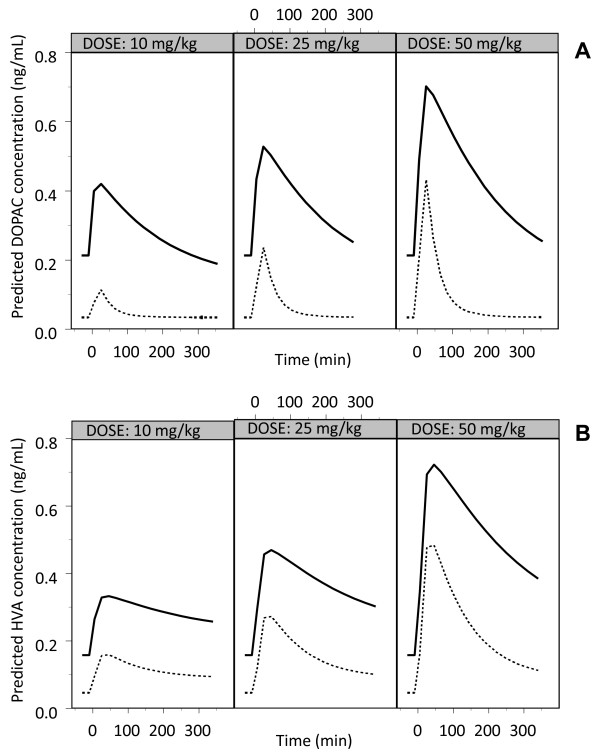
**Population predicted concentrations of DOPAC (panel A) and HVA (panel B) in the control cerebral hemisphere (-) and rotenone-treated responder cerebral hemisphere in responders (---) according to the model as described in Figure 1, at three doses of L-DOPA**.

Striatal microdialysate baseline DOPAC levels in the rotenone-treated responder cerebral hemisphere were about 6 times lower than in the control cerebral hemisphere and averaged 0.2 ± 0.19 pmol/mL and 1.3 ± 0.17 pmol/mL (mean ± SEM), respectively (*p*-value < 0.01; Welch's t-test). Also, striatal microdialysate baseline HVA dialysate levels in the rotenone-treated responder cerebral hemisphere were lower (approximately 4 times) than in the control cerebral hemisphere with respective values of 0.25 ± 0.14 pmol/mL and 0.9 ± 0.08 pmol/mL (mean ± SEM), respectively (*p*-value = 0.02; Welch's t-test). Figure 4 shows the population predicted microdialysate concentrations of DOPAC and HVA versus time for a typical rat per dose group. All structural parameters of the population kinetic model for DOPAC as well as for HVA could be adequately estimated (Table 1). No dose-dependency was found in any of the parameters for DOPAC or HVA. For DOPAC, k46 and k58 (rate constants which describe the conversion of L-DOPA, via dopamine, to DOPAC) do not significantly differ, which means that there appears to be no effect of disease on the metabolism of L-DOPA via dopamine to DOPAC. The same can be said for HVA for which k47 and k59 do not significantly differ. On the other hand, the elimination rate constants were found to be 7-fold and 2.5-fold higher in the rotenone-treated responder compared to the control side for DOPAC (k60 and k80) and HVA (k70 and k90), respectively.

## Discussion

In this study using the rotenone-treated rat as a model for Parkinson's disease, the relationship between plasma and brain_ECF _pharmacokinetics (BBB transport) of L-DOPA and its conversion into the dopamine metabolites DOPAC and HVA, was measured in parallel in the rotenone-treated responder and control cerebral hemisphere at 14 days post-rotenone injection. NONMEM was used to develop a population based pharmacokinetic model. Basal concentrations of DOPAC and HVA in striatal microdialysate were lower in the rotenone-treated responder brain than in the control brain. Furthermore, it was shown that disease-related changes were observed in the kinetics of the dopamine metabolites following L-DOPA administration, without any changes in BBB transport of L-DOPA.

**Figure 5:**
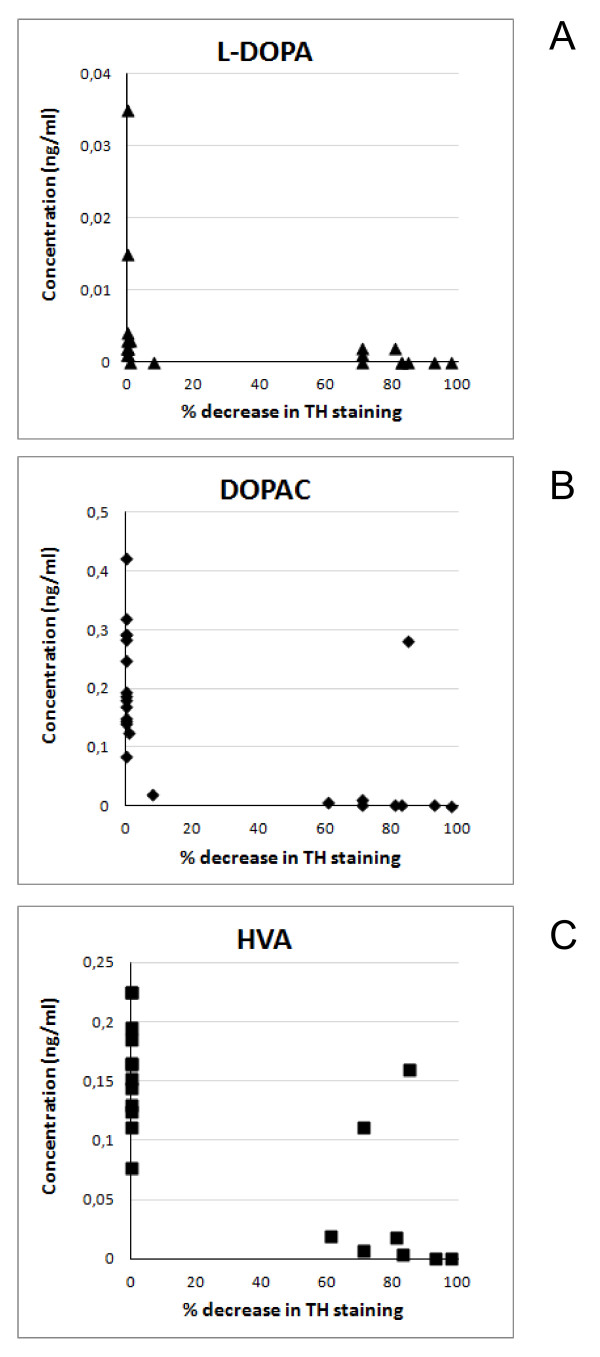
**Individual average baseline concentrations (ng/ml) of L-DOPA (A), DOPAC (B) and HVA (C) as a function of the decrease in TH staining (%)**.

In our study, following the rotenone infusion in the MFB of the rats, the Parkinson's disease state was defined as TH immunostaining in the striatum with a density of less than 40% of control values (termed responders). Data indicated that the number of responders in this model was similar to our previous study [14], and the disease pathology was successfully induced in 70% of the rats. It would be of interest to have more information on potential changes in the pharmacokinetics, BBB transport and conversion of L-DOPA at different stages of Parkinson's disease. In this study the TH% values found at 14 days following rotenone treatment were either close to 100% (non-responders) or smaller than 40% (responders; Figure 5), leaving too small a range to investigate the TH% as a covariate in the model. A shorter interval between rotenone treatment and experiment might have resulted in more diverse values for TH%.

### L-DOPA kinetics in plasma

The results described in this paper are the first in which both plasma and brain_ECF _pharmacokinetics of L-DOPA in control and rotenone-treated conditions are described by a population pharmacokinetic model. The pharmacokinetics of L-DOPA in plasma following intravenous administration was best described by a three-compartmental model, like previously reported [24]. In our study, we were not able to measure endogenous plasma concentrations of L-DOPA (LLQ was 1 ng/mL). Sato *et al*. [25] found in their rat study a basal level of 2.1 ± 0.6 mg/L. In our study, rats were fasted overnight which might be the reason for much lower endogenous L-DOPA in plasma. The total clearance of exogenous plasma of L-DOPA in the study by Sato *et al*. [26] was 3.1 L/h/kg, which is in the same order of magnitude as our value of 6.3 L/h/kg (Cl = 30 mL/min, Table 1; mean weight of the rats before start of the experiment was 288 ± 13 g).

### L-DOPA BBB transport

No difference in BBB transport of L-DOPA between the control and rotenone-treated responder cerebral hemisphere were found. This is in contrast to the findings of an inverse relationship between the MPTP-monkeys' ability to transport L-DOPA from blood to brain and their degree of Parkinsonism by Alexander *et al*. [19]. This may be due to species differences as well as differences in the disease conditions.

### L-DOPA endogenous kinetics

Also, our results did not indicate a disease-induced change in the endogenous production of L-DOPA in the brain (Kin; 5.8 min^-1^). Since dopamine-producing neurons are diminished in Parkinson's disease [4], one would expect a decrease in the endogenous concentration of L-DOPA, as it is a product of the metabolism of L-tyrosine by TH. However, it has been reported that TH enzyme activity, as a function of the degree of dopamine loss, is up-regulated in the striatum of 6-OHDA-lesioned rats and of MPTP-treated rhesus monkeys [27]. This phenomenon was assumed to be a compensatory mechanism of the remaining dopaminergic neurons triggered by the synaptic dopamine loss [28]. It may as well be the case in our situation that up-regulation of TH in the rotenone-treated responder brain is the reason for the absence of disease-induced changes in L-DOPA concentrations in the brain.

### L-DOPA brain metabolism to DOPAC and HVA

The main metabolite of L-DOPA in the early phase after drug administration is dopamine [29,30], which is further metabolized to mainly DOPAC and HVA. The metabolism of L-DOPA via dopamine to DOPAC in the control cerebral hemisphere (0.000044 min^-1^) was indistinguishable from that of the rotenone-treated responder brain (0.000054 min^-1^). Also, the metabolism of L-DOPA via dopamine to HVA in the untreated brain (0.000023 min^-1^) was similar to that in the rotenone-treated responder cerebral hemisphere (0.000016 min^-1^). Furthermore, these values reflect a slow conversion of L-DOPA-via dopamine- to these metabolites. Overall, in contrast to our expectations, it can be said that the disease conditions at 2 weeks post-rotenone-injection into the MFB did not result in any change in the pharmacokinetics of L-DOPA.

### Baseline DOPAC and HVA

The baseline concentrations of DOPAC and HVA were decreased about 10-fold in the rotenone-treated responder brain (Figure 4). These findings are similar to what has been reported in other studies in rats, after an intracerebral injection of rotenone into the MFB [31] and after intracerebral 6-OHDA injection [32,33]. As no changes in formation rate constants of DOPAC and HVA (k46 and k58 for DOPAC, and k47 and k59 for HVA; see Table 1) were found in the rotenone-treated responder relative to the control cerebral hemisphere, a decrease in baseline concentrations results solely from increased elimination rate constants for DOPAC and HVA.

### DOPAC and HVA following administration of L-DOPA

Following L-DOPA administration, differences were found in DOPAC and HVA concentrations and in rate constants for elimination. In our model, the metabolism from dopamine to DOPAC and HVA was explained by first-order kinetics (Figure 1, Table 1), as in the model previously developed [25,26]. Following L-DOPA administration, for DOPAC the elimination rate constant in the rotenone-treated responder cerebral hemisphere (0.038 min^-1^) was increased about 7-fold compared with the elimination rate constant in the control side (0.0053 min^-1^). For HVA this disease-induced increase was about a factor 2.5 (0.011 min^-1 ^for the rotenone-treated responder and 0.0044 min^-1 ^for the control cerebral hemisphere). With unchanged values for the formation rate constants (k46 and k58 for DOPAC, and k47 and k59 for HVA; see Table 1) and lower baseline concentrations measured for both DOPAC and HVA, the higher elimination rate constant as found for DOPAC and HVA would be possible if dopamine concentrations were lower in the rotenone-treated responder cerebral hemisphere such that metabolite formation rate-dependent elimination occurs. This is also called "flip-flop kinetics" [34], i.e. [metabolite formation rate constant × amount of metabolite remaining to be formed] is about equal to the [metabolite elimination rate constant × amount of metabolite remaining to be eliminated]). Reduced dopamine concentrations in the rotenone-treated responder cerebral hemisphere are indeed plausible with a diminished amount of dopaminergic neurons as indicated by substantially decreased TH staining.

A number of processes may contribute to the elimination of DOPAC and HVA from the brain. Elimination of DOPAC may occur by conjugation, in rats mostly to sulphates [35], or by transformation to HVA by catechol-O-methyltransferase. For HVA, formed as metabolite of DOPAC but also directly from dopamine [36-38], the mechanisms of elimination from the brain are not fully clear. HVA may leave the brain by passive diffusion [38]. Also, HVA leaves the brain via a probenecid-sensitive organic anion transport (OAT3) system, present at the BBB [39]. This HVA efflux transport system is likely to play an important role in controlling the level of HVA in the brain. An apparent *in vivo *efflux rate constant of HVA from the brain was determined by Mori *et al*. [36] and found to be 0.017 min^-1^. Our value for the elimination rate constant of HVA in the control cerebral hemisphere was 0.044 min^-1 ^and of the same order of magnitude.

Although the expression of OATs is affected (mainly down-regulated) in certain renal and hepatic diseases [40], to our knowledge, no studies have been performed indicating changes in OAT functionality at the BBB. The increased values that we found for the elimination rate constants for DOPAC and HVA could have been the result of up-regulation of one or more of active elimination processes that we are not yet able to specify.

In general, the integrated approach used in this study, including plasma pharmacokinetics, BBB transport and *in vivo *assessment of dopamine system functionality at different stages of the disease may help to unravel more mechanistically the factors that play a role in effective Parkinson's disease treatment.

## Conclusions

The developed population pharmacokinetic model allowed the integration of the kinetics of L-DOPA and its conversion to DOPAC and HVA in both control and rotenone-treated responder brain. It was demonstrated that 2 weeks following a unilateral infusion of rotenone into the rat brain, dopamine depletion resulted in lower brain levels and higher elimination rates of DOPAC and HVA for the diseased cerebral hemisphere. This was, however, not accompanied by changes in the plasma pharmacokinetics and BBB transport of L-DOPA. Taken together, the varying results on whether or not changes in BBB transport in Parkinson's disease in previous studies and in this study indicate that changes in BBB functionality are not specifically associated with Parkinson's disease, and therefore cannot account for the decreased benefit of L-DOPA at later stages of Parkinson's disease.

## List of abbreviations

BBB: blood-brain barrier; DOPAC: 3,4-dihydroxyphenylacetic acid; HVA: homovanillic acid; L-DOPA: 3,4-dihydroxy-L-phenylalanine; TH: tyrosine hydroxylase; MFB: medial forebrain bundle.

## Competing interests

The authors declare that they have no competing interests.

## Authors' contributions

PR carried out the experiments, modelling of the data and contributed to writing of the manuscript, HD contributed to the model development and writing of the manuscript, MO contributed to the staining of the brain tissues, interpretation of the data and writing of the manuscript, MD participated in the design of the study and revision of the manuscript, EL conceived the project of which this study was a part of, participated in design and coordination of the study, and contributed to writing of the manuscript. All authors have read and approved the final version of the manuscript.
